# Viable but nonculturable state in the zoonotic pathogen *Bartonella henselae* induced by low-grade fever temperature and antibiotic treatment

**DOI:** 10.3389/fcimb.2024.1486426

**Published:** 2024-11-21

**Authors:** Yuze Gou, Dongxia Liu, Yuxian Xin, Ting Wang, Jiaxin Li, Yiwen Xi, Xiaoling Zheng, Tuanjie Che, Ying Zhang, Tingting Li, Jie Feng

**Affiliations:** ^1^ Key Laboratory of Preclinical Study for New Drugs of Gansu Province, School of Basic Medical Sciences, Lanzhou University, Lanzhou, China; ^2^ State Key Laboratory of Veterinary Etiological Biology, College of Veterinary Medicine, Lanzhou University, Lanzhou, China; ^3^ Department of Scientific Experimental Research, Innovation Center of Functional Genomics and Molecular Diagnostics Technology of Gansu Province, Lanzhou, China; ^4^ State Key Laboratory for the Diagnosis and Treatment of Infectious Diseases, The First Affiliated Hospital, Zhejiang University School of Medicine, Hangzhou, China; ^5^ Center for Microbiome and Disease Research, Jinan Microecological Biomedicine Shandong Laboratory, Jinan, China

**Keywords:** *Bartonella*, *B. henselae*, VBNC, resuscitation, persister, blood-culture-negative endocarditis (BCNE), cat-scratch disease (CSD)

## Abstract

The zoonotic pathogen *Bartonella henselae* is responsible for diverse human diseases, from mild to life-threatening, but it often eludes detection in culture-based assays. This study investigates the potential of *B. henselae* to enter a viable but nonculturable (VBNC) state when exposed to human fever temperature or antibiotics, with this state confirmed by successful resuscitation. Viability was assessed using SYBR Green I/PI staining and propidium monoazide–quantitative polymerase chain reaction (PMA-qPCR), while culturability was determined through colony-forming unit (CFU) counting on blood agar plates. Resuscitation of VBNC cells was attempted using modified Schneider’s medium with 10% defibrillated sheep blood. In the results, *B. henselae* cells entered a VBNC state after 19 days of exposure to 38.8°C. Antibiotics, particularly with bactericidal activity, induced the VBNC state within 4 days treatment. Successful resuscitation confirmed the VBNC state developed via the above two strategies. Transmission electron microscopy (TEM) examination revealed intact cell structures and dense cytosol in VBNC cells, with a significant increase in plasmolytic cells. Notably, VBNC cells demonstrated greater drug tolerance than cells in the stationary phase, which encompassed a substantial portion of persisters. Proteomic analysis revealed the up-regulation of proteins linked to host cell invasion and stress resistance, while proteins related to signaling and cellular processes were down-regulated. Fluorescence *in situ* hybridization (FISH) analysis confirmed that the VBNC state truly boosted *B. henselae*’s invasion of HUVECs. This study highlights *B. henselae*’s capacity to enter a VBNC state under thermal and antibiotic stress, emphasizing the urgent need for advanced diagnostic and therapeutic strategies to effectively target VBNC cells, which complicate diagnosis and treatment.

## Introduction

Bartonellae are fastidious vector-borne Gram-negative bacteria responsible for multiple human diseases (Jin et al., 2023). The predominant Bartonelloses in humans are primarily attributed to *Bartonella henselae*, *Bartonella bacilliformis*, and *Bartonella quintana*. Infection with *B. henselae* can result in the prototypical cat-scratch disease (CSD), characterized by fever and regional lymphadenopathy, primarily affecting immunocompromised individuals and occasionally immunocompetent individuals, especially children and adolescents (Pulliainen and Dehio, 2012). In severe cases, *B. henselae* can lead to bacillary angiomatosis, a condition marked by vasoproliferative tumors affecting the skin and internal organs (Pulliainen and Dehio, 2012). Additionally, a broad spectrum of manifestations has been reported in association with persistent infection of *B. henselae*, including asymptomatic bacteremia, which is a distinctive characteristic of *Bartonella* (Massei et al., 2004; Breitschwerdt et al., 2008). The prevalence of *B. henselae* infection is worldwide, with a seroprevalence in China of up to 9.68% (Song Xiu et al., 2020). In the United States, there are estimated 12,500 cases of CSD annually, with the proportion of hospitalized CSD patients increasing from 3.5% in 2005–2007 to 4.2% in 2011–2013 (Nelson et al., 2016). Despite the prevalence, diagnosing *B. henselae* infections remains challenging. Antibody-based serological assays are widely applied for diagnosing *B. henselae* infection. However, these assays present several limitations, including cross-reactivity with other species and organisms, variability in antigen preparations, cutoff titers, and other factors (La Scola and Raoult, 1996; Massei et al., 2004).

Traditional diagnostic methods based on bacterial cultivation (Drummond et al., 2023; Koutantou et al., 2023), often face challenges due to the fastidious nature of *B. henselae*, which is difficult to culture and detect. This limitation can result in diagnostic difficulties, as in blood-culture-negative endocarditis (BCNE) (Okaro et al., 2017). While molecular techniques offer higher detection rates (Prudent et al., 2018; Sayed et al., 2022; Koutantou et al., 2023), they do not confirm the presence of live bacterial cells. Despite the well-established causative relationship between *B. henselae* and certain diseases, the difficulty of isolating *B. henselae* from some infected hosts, combined with the limitations of molecular detection methods, means that these techniques alone cannot fully satisfy Koch’s postulates. This difficulty underscores the importance of microbial culture, still considered the gold standard for detecting infectious pathogens. Higher recovery rates from clinical specimens have been achieved by the shell-vial technique, which involves the inoculation of clinical specimens onto confluent cell monolayers on coverslips within shell vials, followed by low-speed centrifugation to enhance bacterial attachment and penetration, with subsequent detection via immunofluorescence, and definitive bacterial identification by PCR (Lagier et al., 2015). In a study of endocarditis patients suspected of *Bartonella* spp. infection, the recovery rate was 44% using a shell vial culture assay, compared to only 4% when directly culturing on Columbia sheep blood agar plates (La Scola and Raoult, 1999). The improved sensitivity for recovering *Bartonella* isolates by shell-vial culture methods suggests that certain factors in the subculture system are essential for the growth of some *Bartonella* isolates, which may enter a viable but nonculturable (VBNC) state and have the potential to revert to a culturable state under appropriate conditions.

The VBNC state is a survival strategy employed by various bacterial species, allowing them to maintain cellular integrity and metabolic activity while remaining undetectable by standard culturing methods (Liu et al., 2023). VBNC bacteria can resuscitate under favorable conditions, potentially leading to recurrent infections and complicating treatment. For example, VBNC *Listeria monocytogenes* can resuscitate and cause listeriosis in vulnerable populations (Lotoux et al., 2022). VBNC cells also exhibit resistance to antibiotics and immune stress (Ayrapetyan et al., 2015b), contributing to persistent infections. The persistence of VBNC pathogens in hosts, especially intracellular pathogens like *Bartonella*, poses significant challenges for accurate diagnosis and risk assessment in clinical settings.

Given these challenges, this study aimed to determine whether *B. henselae* can enter a VBNC state and to characterize these cells in detail. We induced the VBNC state in *B. henselae* using high temperature and antibiotic treatment, mimicking conditions like fever and antimicrobial exposure, and developed a method for resuscitating *B. henselae* VBNC cells, which could greatly improve the diagnosis, detection, and treatment of bartonellosis.

## Materials and methods

### Bacterial strain and culture conditions

*Bartonella henselae* strain Houston-1 (ATCC 49882) was cultured in a modified Schneider’s medium (Riess et al., 2008; Li et al., 2019). Briefly, 10 mL modified Schneider’s medium was composed of 8 mL Schneider’s drosophila medium (Gibco, USA), 1 mL heat-inactivated fetal bovine serum (VivaCell, China), and 1 mL 50% sucrose solution. Cultures were incubated at 33°C without shaking. Colony forming units (CFU) were counted after serial 10-fold dilutions on Columbia blood agar plates with 5% defibrinated sheep blood, followed by incubation at 33°C.

### Quantification of live *B. henselae* cells via SYBR Green I/PI assay

The viability of *B. henselae* was evaluated using a SYBR Green I/propidium iodide (PI) staining method (Feng et al., 2014; Li et al., 2019). Bacterial samples were stained with SYBR Green I (Invitrogen, USA) and PI (Solarbio, China), incubated in darkness at room temperature (RT) for 30 minutes, and counted under an IX71 inverted microscope (Olympus, Japan). Dead cells, marked by red fluorescence emitted from PI, were distinguished from live cells, which exhibited green fluorescence due to SYBR Green I binding.

For plate reader analysis, standard samples were prepared by mixing live and 70% isopropanol-killed bacteria in varying ratios. Both these standards and culture samples were stained with SYBR Green I/PI, incubated at RT in darkness for 30 minutes, and analyzed using a fluorescence plate reader. Green and red fluorescence intensities were measured using a fluorescence plate reader (Thermo Fisher Scientific, USA). The percentage of viable *B. henselae* cells in each sample was calculated based on the ratios of green to red fluorescence intensities, providing a quantitative assessment of cell viability under various experimental conditions.

### Quantification via PMA-qPCR

Live *B. henselae* cells were quantified using the PMA-qPCR method, following a previously described protocol (Lazou et al., 2019). Briefly, 990 μL aliquots of bacterial cultures were added with 10 μL propidium monoazide (PMA) (Bioscience, China) in Eppendorf tubes and kept at RT for 10 min in darkness with occasional mixing. Samples were then exposed to light for 15 min, followed by centrifugation at 10,000 g for 5 min. DNA was extracted from the cell pellets using QIAamp DNA Mini Kit (Qiagen, Germany). For the calibration standard curve preparation, mixtures of live and 3% H_2_O_2_-killed bacteria were prepared to create five different ratios of viable cells. These mixtures were treated in parallel with the samples described above. The extracted DNA from each sample, including standards and samples, was mixed with Hieff UNICON Universal Blue qPCR SYBR Green Master Mix (YEASEN, China) and specific primers (Bhe-16s-qF AATCTTGCGACCGTACTCCC, Bhe-16s-qR TCCACGCCGTAAACGATGAA) targeting the *B. henselae* 16S rRNA gene. Quantitative PCR was performed on a QuantStudio (Applied Biosystems). Live *B. henselae* cells were quantified using a PMA-qPCR method (Lazou et al., 2019). The PMA inhibition factor was calculated using the formula 2^(Ct(PMA treated)-Ct(PMA untreated)), and the viability percentage was determined by 100/(PMA inhibition factor) and calibrated using the standard calibration curve.

### Induction of VBNC cells

VBNC cells were induced by two methods: culture under fever-like temperature (38.8°C) up to 25 days and treatment with antibiotics at 37°C. The antibiotics tested included gentamicin (15 μg/mL), streptomycin (43 μg/mL), doxycycline (7 μg/mL) and erythromycin (1.44 μg/mL). Cultures were periodically sampled to assess viability and culturability. The drugs were prepared according to the manual of clinical and laboratory standards institute (CLSI) and sterilized with a 0.22 μm filter. Methylene blue was dissolved in DMSO.

### Resuscitation of *B. henselae* from the VBNC state

Cultures of *B. henselae* cells in the VBNC state-inducing course were periodically drawn. Samples were then divided into two aliquots. One aliquot from each sample was directly deposited onto blood agar plates, and the second aliquot underwent a resuscitation process. For the aliquot dropped on the blood agar plate, drug removal and resuspension were required for antibiotic-induced VBNC cells in the culture. For the resuscitation, cells were collected by centrifugation (5000×g, 5 min), resuspended in the modified Schneider’s medium supplemented with 10% defibrinated sheep blood, and incubated at 33°C for 1 day, followed by plating on Columbia blood agar plate. The successful resuscitation of *B. henselae* in pure VBNC state was achieved when the aliquot gone through resuscitation displayed colonies but the parallel aliquot directly deposited on blood agar plates failed to do so.

### Transmission electron microscopy

Bacterial cells were pelleted and fixed with 2.5% glutaraldehyde in 0.1 M sodium cacodylate buffer. After that, the cells were suspended in 2% agarose and cut into cubes. Then, samples were washed and resuspended in 0.1 M sodium cacodylate buffer, and fixed in osmium tetroxide. After staining, samples were washed, dehydrated, and soaked in acetone, followed by immersion in 3:1 acetone: epoxy resin, 1:1 acetone: epoxy resin and pure epoxy resin overnight. Finally, samples were cut into ultrathin sections, stained with uranyl acetate and lead citrate, and visualized with a Hitachi HT7700 transmission electron microscope. Cell size and shape were analyzed using Fiji software (Schindelin et al., 2012).

### DIA (data-independent acquisition) proteomic analysis by LC-MS/MS

*B. henselae* cells were pelleted by centrifugation and washed with PBS to remove residual culture medium. Cell pellets were resuspended in lysis buffer composed of 4% SDS, 100 mM DTT, and 100 mM Tris-HCl (pH 8.0), boiled for 3 min, and were then ultrasonicated, with insoluble cellular debris removed after centrifugation at 16,000 g for 15 min. The resultant clear supernatant was then assayed for protein concentration using the BCA Protein Assay Kit (BeyoTime, China). Protein digestion was conducted by adopting the Filter-Aided Sample Preparation (FASP) technique. LC-MS/MS analyses were performed with an Orbitrap Astral mass spectrometer linked to a Vanquish Neo UHPLC system. Peptides were loaded into a Low-Load µPAC™ Neo HPLC Column (Thermo Scientific). The mobile phase consisted of (A) water with 0.1% formic acid, and (B) 80% acetonitrile with 0.1% formic acid. We used a DIA method that included a survey scan from 380-980 m/z at resolution 240000 with AGC target of 500% and 5 ms injection time. The energy and timing for the scans were precisely controlled, and the resulting spectra were recorded in two different formats. The DIA MS/MS scans were acquired by Astral from 150-2000 m/z with 2 m/z isolation window and with AGC target of 500% and 3 ms injection time. The spectra of full MS scan and DIA scan were recorded in profile and centroid type, respectively. Data were analyzed using the DIA-NN 1.8.1 (Demichev et al., 2020), and underwent a database search against the GenBank database, specifically targeting the NCBI for *B. henselae* Houston-1 sequence (NC_005956). Results from the database search were refined to ensure a false discovery rate of less than 1% at both the peptide-spectrum match and protein levels. Gene ontology enrichment analysis, heat map figure and volcano figure were executed by using KEGG database (Kanehisa et al., 2017) and statistical language R with ggplot2, pheatmap and EnhencedVolcano packages.

### Fluorescence *in situ* hybridization of HUVECs

Prior to infection, log-phase *B. henselae* cultured at 33°C and *B. henselae* VBNC induced by culturing at 38.8°C for 19 days were washed twice with PBS and resuspended in DMEM (F12) medium. Human umbilical vein endothelial cells (HUVECs, CCTCC GDC0635) were washed twice with PBS before being infected with a multiplicity of infection (MOI) of 100 of *B. henselae* either in logarithmic phase or in VBNC state. The plates were centrifuged at 1800 g for 5 min and then incubated in 5% CO_2_ at 37°C or 38.8°C. At various post-infection time points, the plates were washed thoroughly with PBS and fixed with 4% paraformaldehyde for 2 h. Following fixation, cells were washed three times with PBS, and then treated with PBS containing 0.1% Triton X-100 for 10 min to permeabilize the cell membrane. Cells were then dehydrated in 50%, 80%, and 100% ethanol, respectively, and hybridized overnight with the BA23 probe (5’-6-FAM-CTATCACCCTCTTTGGTTCG-3’) in hybridization buffer (900 mM NaCl, 20% formamide, 0.01% SDS, 20 mM Tris, pH8.0) (Prudent et al., 2017). After hybridization, the cells were washed with washing buffer (0.225 M NaCl, 5 mM EDTA, 0.01% SDS, pH 8.0) and then stained with DAPI for 3 min. Following three washes with ddH_2_O, coverslips were sealed and examined using a Zeiss LZM 900 confocal laser scanning microscope.

## Results

### VBNC formation at elevated temperature

**Figure 1:**
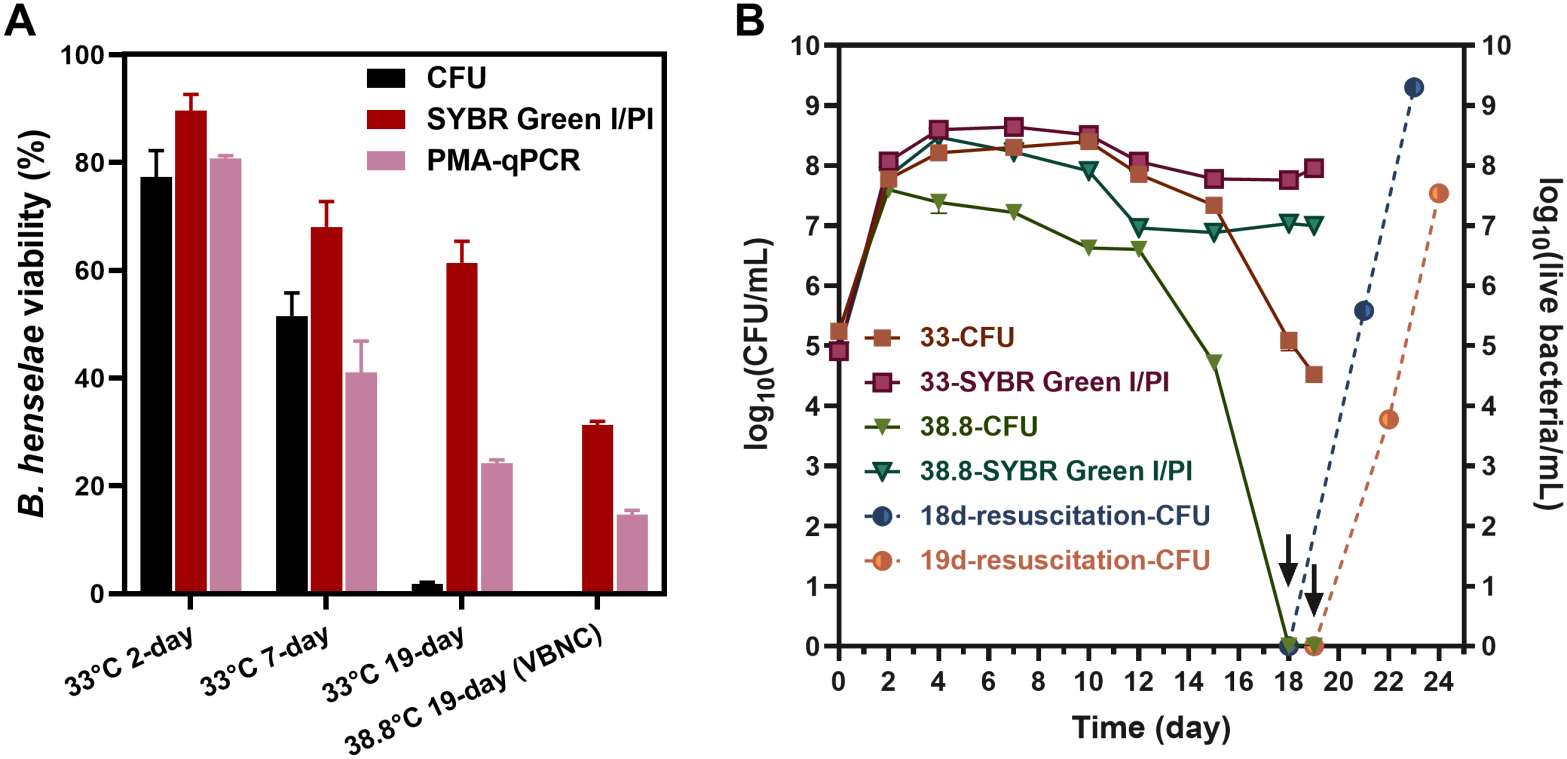
Temperature effect on the formation of *B. henselae* VBNC cells. **(A)** Evaluation of culturability and viability for *B. henselae* cells cultured at 33°C and 38.8°C, respectively. Culturable cells were monitored by CFU counting, whereas viable cell counting were monitored by SYBR Green I/PI assay and PMA-qPCR assay. The CFU-based viability for each sample was determined by dividing the CFU count by the total cell count observed under the microscope. Similarly, the SYBR Green I/PI-based viability was calculated by dividing the count of green-stained (live) cells by the total cell count under the microscope. For the PMA-qPCR assay, viability was calculated based on the PMA inhibition factor and calibrated by the calibration curve. **(B)** The entry of VBNC state at 33°C and 38.8°C were monitored over time. The arrows and dash lines represent the resuscitation for 18-day-cultured and 19-day-cultured VBNC bacteria at 38.8°C. All tests were run in triplicates, and all data are averages of biological triplicates ± standard deviation.

We investigated the impact of temperature on the formation of VBNC cells by assessing cell viability and culturability of *B. henselae* at 38.8°C and 33°C. Culturable cells were determined by CFU counting on blood agar plates, while viable cells were evaluated using SYBR Green I/PI staining and/or PMA-qPCR. The viability measurements from these methods showed a strong linear relationship (**Supplementary Figure S1**). At 33°C, *B. henselae* cells remained viable from the 2-day logarithmic phase to the 19-day late-stationary phase. However, culturability significantly declined, from 77.3% in 2-day cultures to 1.8% in 19-day cultures (**Figure 1A**), indicating VBNC cell formation. At 38.8°C, approximately 20%-30% of the cells remained viable after 19 days, but no colonies formed on blood agar plates (**Figure 1A**), suggesting that the entire population of viable cells had entered a VBNC state.

Overtime, at 33°C, viable and culturable cells were initially comparable, but culturable cells declined markedly by 18- and 19-day, confirming VBNC cell presence. At 38.8°C, viable cells remained relatively constant, but no culturable cells were detected by 18- and 19-day, confirming a pure VBNC state. Resuscitation experiments demonstrated that *B. henselae* could recover from the VBNC state (**Figure 1B**).

### Morphological and physiological changes in *B. henselae* VBNC cells

**Figure 2:**
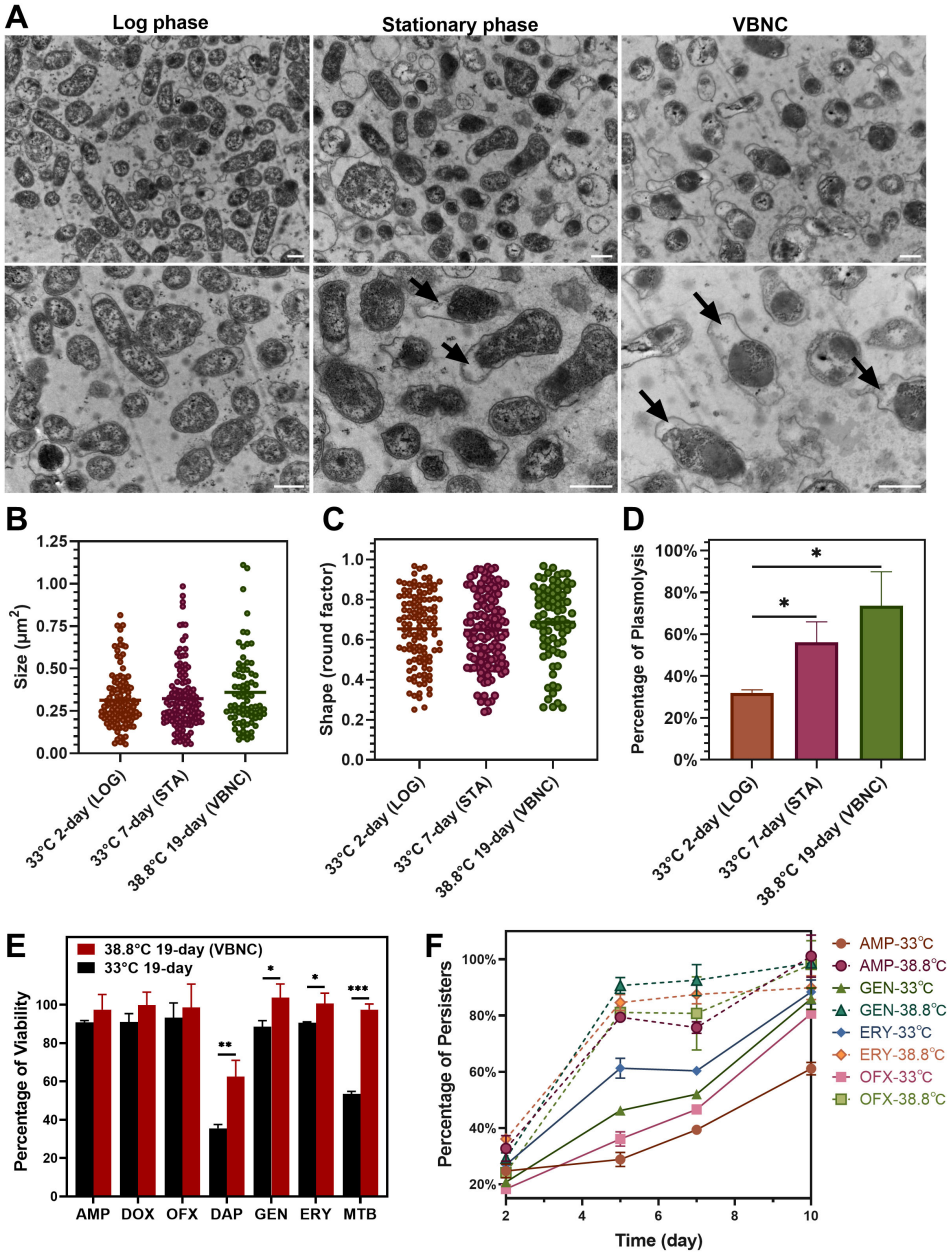
Comparison of morphology and drug tolerance of *B. henselae* cells across different states. **(A)** TEM images show the morphology of *B. henselae* in the logarithmic phase (LOG), stationary phase (STA), and VBNC state, with a scale bar of 0.5 μm. ImageJ software was used to analyze bacterial cell size **(B)**, shape **(C)** and the percentage of plasmolysis **(D)**. **(E)** The percentage of viable cells in the population after drug exposure. *B. henselae* cells were separately treated for 10 days with different drugs, including clinically used (ampicillin, doxycycline, ofloxacin, gentamicin, erythromycin) and anti-persister drugs (daptomycin, methylene blue). Over 15-fold MIC for each antibiotic (100 µg/mL) was used to sort out persisters from bacterial populations in cultures. Viable cells were quantified using SYBR Green I/PI staining combined with a plate reader assay. **(F)** The formation of *B. henselae* persisters was monitored over time at 38.8°C and 33°C. Statistical significance between groups was assessed using *t-*tests, with significant differences indicated by asterisks (*P <0.05; **P <0.01; ***P <0.001).

Bacteria typically undergo a series of changes in morphological structure and physiological characteristics upon entering the VBNC state. In this study, TEM was utilized to observe the morphological adjustments and intracellular content changes in *B. henselae* cells in the VBNC state. We observed that VBNC cells maintained intact cell walls, membranes and abundant intracellular content, with no significant changes in shape or size (**Figures 2A–C**). However, many VBNC cells exhibited plasmolysis (**Figures 2A, D**), a morphological abnormality characterized by the cell membrane shrinking away from the cell wall, especially when compared to cells in the logarithmic and stationary phases. The intracellular contents in plasmolytic VBNC cells appeared to be more intensely condensed relative to those in cells from the other two cultures, with a pronounced separation between the cell membrane and the cell wall. These observations suggest that while *B. henselae* VBNC cells retain their overall structural integrity they undergo significant intracellular condensation and membrane detachment, indicative of a stress response and metabolic adjustment characteristic of the VBNC state.

### Drug tolerance of *B. henselae* VBNC cells

VBNC cells of various bacterial species exhibit robust competence in surviving antibiotics of multiple classes. We compared the drug susceptibility of VBNC cells cultured at 38.8°C for 19 days with cells cultured at 33°C for 19 days. VBNC cells showed significant tolerance to multiple antibiotics, especially anti-persister drugs like daptomycin and methylene blue (**Figure 2E**). Meanwhile, the corresponding MIC values for the resuscitated VBNC cells were identical to those of logarithmic-phase cells (**Supplementary Table S1**), consistent with previous suggestion that VBNC cells are part of drug-tolerant persisters, not drug-resistant mutants (Zhang, 2014; Ayrapetyan et al., 2015a). Notably, over 80% of *B. henselae* cells at 38.8°C developed multi-drug tolerance within just 5 days’ culture, while cells at 33°C took 10 days to reach similar levels (**Figure 2F**). The enhanced development of drug-tolerant VBNC cells of *B. henselae* at elevated temperatures poses a significant treatment challenge, particularly under febrile conditions.

### Antibiotic-induced VBNC state in *B. henselae*

**Figure 3:**
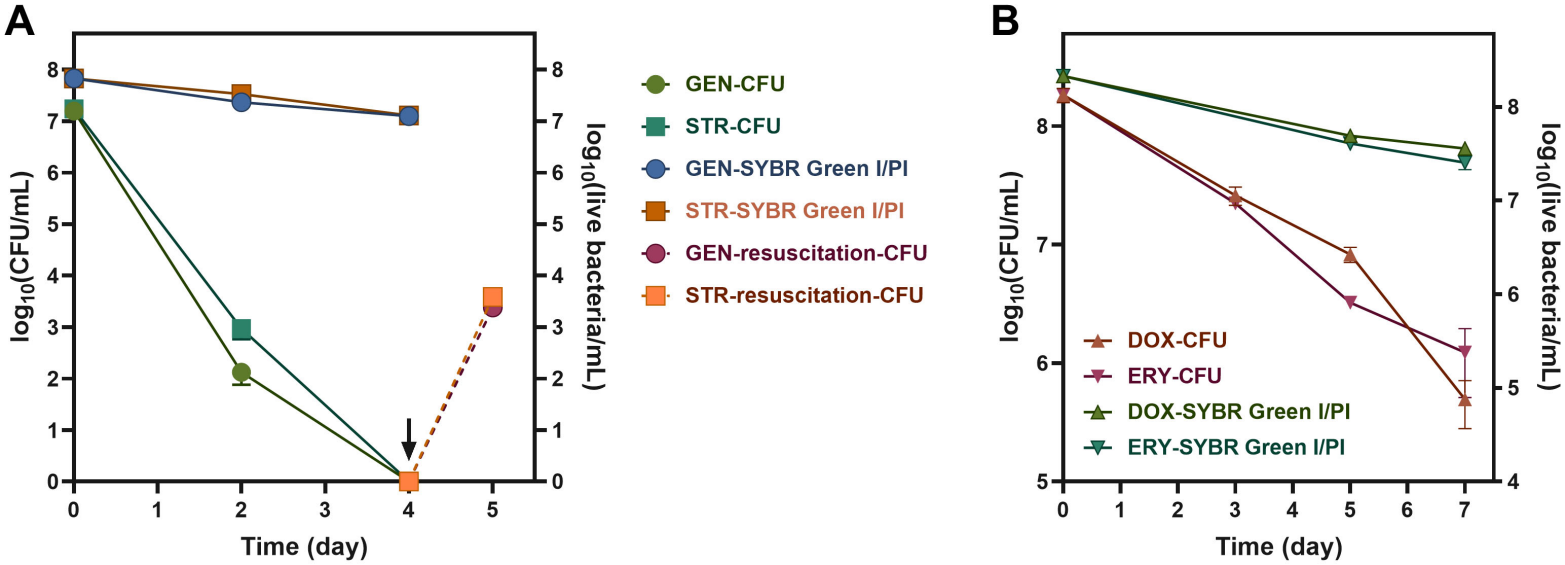
Antibiotic induction of *B. henselae* into the VBNC state. The culturability and the viability curve of *B. henselae* treated with bacteriocidal antibiotics **(A)** and bacteriostatic antibiotics **(B)** at 37°C. The arrows and dash lines represent the resuscitation of VBNC cells. The culturable cells and viable cells were monitored by CFU and SYBR Green I/PI assay, respectively. All tests were run in triplicates, and all data are averages of biological triplicates ± standard deviation. GEN, gentamicin; STR, streptomycin; DOX, doxycycline; ERY, erythromycin.

To explore if antibiotics could also induce VBNC states, we treated *B. henselae* cultures with several clinically used antibiotics at their maximum serum concentrations, defined as the highest concentrations achievable in human serum following administration. Treatment with gentamicin and streptomycin for 4 days led to a slight decrease of viable cells and undetectable levels of culturable cells (**Figure 3A**), which indicates that *B. henselae* fully transitioned into the VBNC state, as confirmed by successful resuscitation. In contrast, doxycycline and erythromycin led to only a partial transition to the VBNC state after 7 days (**Figure 3B**), indicating that bactericidal antibiotics (gentamicin and streptomycin) are more effective in inducing the VBNC state than bacteriostatic agents.

### Divergent proteomic profiles between VBNC and culturable *B. henselae* cells

**Figure 4:**
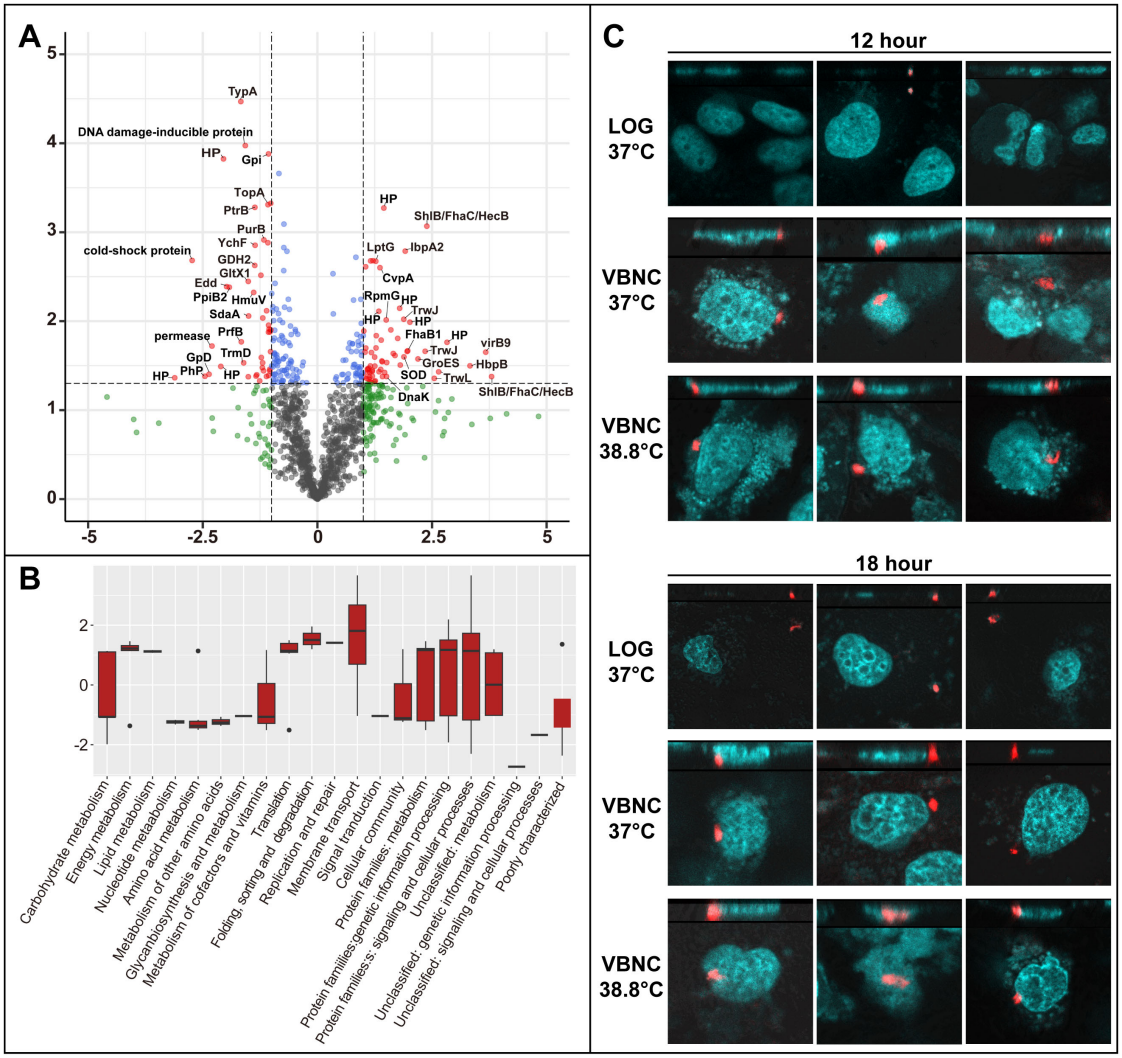
Differential protein expression in *B. henselae* VBNC cells and their ability to invade HUVECs. **(A)** Comparison of protein expression in VBNC cells *vs*. late-stationary-phase cells. A volcano plot showes significant protein expression changes in VBNC cells (*p*-value < 0.05, log_2_FC ≥ 1 or log_2_FC ≤ -1) marked in red. Other proteins with less pronounced changes are shown in green (log_2_FC ≥ 1 or log_2_FC ≤ -1, but *p*-value ≥ 0.05) and blue (*p*-value < 0.05, but log_2_FC between -1 and 1). Protein label abbreviations: HP, hypothetical protein; PhP, phage protein. **(B)** A bar chart depicts the enrichment of Gene Ontology terms among significantly altered proteins (*p*-value < 0.05, log_2_FC ≥ 1 or log_2_FC ≤ -1), categorized by KEGG pathways and visualized using R ggplot2 boxplots. For **(A, B)**, VBNC cells were confirmed through a resuscitation assay after 19 days of incubation at 38.8°C, with the control group of *B. henselae* cells cultured at 33°C for 19 days. **(C)** The invasion of HUVECs by *B. henselae* VBNC cells and cells at the logarithmic stage was analyzed at 12 and 18 hours post-infection using FISH combined with confocal scanning microscopy. A multiplicity of infection (MOI) of 100 of *B. henselae* was used either in logarithmic phase or in VBNC state. *B. henselae* cells, labeled with a specific 16S rRNA probe, appear in red, while HUVECs nucleic acids, stained with DAPI, are shown in cyan.

To understand the molecular mechanisms governing the VBNC state, we conducted a comparative proteomic analysis between VBNC cells and late-stationary-phase cells. The datasets have been deposited to the iProX partner repository (Chen et al., 2022) with the identifier PXD055316. Out of 1230 identified proteins, 217 showed significant differential expression (*p*-value < 0.05). Of these, 104 proteins were up-regulated (FC >= 1.5), with 63 exhibiting FC values exceeding 2.0, while 113 were down-regulated (FC <= 0.67), with 45 falling below 0.5 (**Figure 4A**). Particularly, up-regulated expression of HecB/FhaC family proteins (BH07920, BH06540 and BH06660), filamentous hemagglutinin (FhaB1), the hemin-binding protein (BH02570), TrwL/J, VirB, invasion-associated locus B (IalB), BafA, and superoxide dismutase (SOD) was observed. *B. henselae* VBNC cells also up-regulated molecular chaperones and heat shock proteins, including GroES (HSP10 family), GroEL (HSP60 family), DnaK (HSP70 family), and IbpA (HSP20 family).

Pathways related to energy and lipid metabolism were notably up-regulated, while those associated with nucleotide, amino acid, glycan, cofactor, and vitamin metabolism were down-regulated (**Figure 4B**), reflecting the metabolic adaptation of VBNC cells.

### Rapid invasion of HUVECs by VBNC *B. henselae*

Proteomic analysis revealed that VBNC *B. henselae* upregulates proteins related to adhesion and host cell invasion. Using FISH and confocal scanning microscopy, we found that VBNC cells rapidly invade HUVECs, with internalization observed within 12-18 hours post-infection, whereas no internalization was observed with logarithmic-phase *B. henselae* at the same time points (**Figure 4C**).

## Discussion

Microbial cultivation has long been considered the gold standard for identifying pathogens in clinical samples. However, the existence of VBNC bacteria, which do not grow on routine culture media, complicates clinical diagnosis. For *Bartonella* species, cultivation on blood agar plates is critical for diagnosing infections (La Scola and Raoult, 1999; Okaro et al., 2017). However, cases of “culture-negative” endocarditis caused by *B. henselae* have been reported (Okaro et al., 2017). Furthermore, culture-based tests fail to detect all *Bartonella* in blood samples, with only 69% (11/16) of *Bartonella-*infected samples successfully cultured (Pitassi et al., 2015). Shell-vial culture techniques have improved the recovery rates of *Bartonella* from clinical specimens (Lagier et al., 2015), particularly compared to direct culture on blood agar plates (La Scola and Raoult, 1999), suggesting that *Bartonella* spp. may enter a viable but nonculturable (VBNC) state under certain conditions, which could be one of the causes for the failure in cultivation.

To prove that *B. henselae* can enter a VBNC state under specific conditions, *B. henselae* was cultivated at 38.8°C, a temperature equivalent to the body temperature of cats and dogs and the fever temperature in humans, to induce the VBNC state in this study. We found that *B. henselae* could enter a pure VBNC state after 19 days at 38.8°C, with no culturable cells detected (**Figure 1B**). The formation of VBNC cells was primarily indicated by discrepancies between the number of culturable cells and the total viable cell count. Culturable cells were routinely quantified through CFU counts on agar plates, while total viable cells were assessed using cell membrane integrity-based methods, such as PMA-qPCR (Kibbee and Ormeci, 2017; Lazou et al., 2019) and SYBR Green I/PI staining (Feng et al., 2018). The viability percentages measured by PMA-qPCR showed a linear correlation with those obtained using SYBR Green I/PI staining combined with plate reader or microscopic analysis (**Supplementary Figure S1**). However, PMA-qPCR consistently reported lower viability compared to SYBR Green I/PI staining combined with microscopic observation (**Figure 1A**), indicating a higher signal from dead bacterial cells in PMA-qPCR. It is well-documented that extracellular DNA can lead to overestimations of viable bacterial counts in PMA-qPCR (Lovdal et al., 2011). Although washing bacterial cells may mitigate this bias, the additional washing and centrifugation steps can damage fragile *B. henselae* cells, further complicating the accurate assessment of viability. In this study, SYBR Green I/PI staining was favored for its faster and convenient sample processing, eliminating the need for cell washing and pelleting, and its minimal sample requirements (10 μL), making it an ideal method for the timely assessment of resuscitation.

Clinically used antibiotics, particularly bactericidal ones like gentamicin and streptomycin, also triggered the VBNC state (**Figure 3**), converting the entire viable population into VBNC state after just 4 days. Over 100 bacterial species have been documented to enter a nonculturable state, with some species, such as *Mycobacterium tuberculosis* (Zhang et al., 2001), *Vibrio vulnificus*, and *Legionella pneumophila*, can resuscitate (Li et al., 2014). Given resuscitation is now a gold standard for verifying the VBNC state (Liu et al., 2023), we developed a specialized medium as described in the Methods section and successfully resuscitated VBNC cells generated by cultivation at 38.8°C or antibiotic treatment, which otherwise failed to grow on regular blood agar plates. The resuscitation method developed here would be promising to improve the diagnostic detection of *B. henselae* in clinical practice, though further studies in clinical settings are needed.

The viability assessment of bacterial cells in this study (PMA-qPCR assay and SYBR Green I/PI assay) relied on membrane integrity, which might misclassify dead cells with intact membrane as VBNC cells (Song and Wood, 2021). However, TEM analysis confirmed that *B. henselae* VBNC cells developed at 38.8°C maintain an intact cell wall, intact inner membrane, and dense cytosol (**Figure 2**). Notably, substantial shrinkage of the inner membrane away from the cell wall was observed in a large portion of VBNC cells, highlighting its relevance under stress conditions. Similar morphology was reported in *E. coli* in response to nutrient depletion (Shi et al., 2021), and the presence of plasmolysis implies that bacterial cells were alive (Korber et al., 1996). The morphology changes of VBNC *B. henselae* need further study in the future.

The ability of *B. henselae* to enter the VBNC state likely contributes to the frequent failure to isolate the bacterium from hosts, posing challenges for clinical diagnosis and treatment. The generation of a pure VBNC state at an elevated temperature of 38.8°C in our study suggests that *B. henselae* may exist in the VBNC state within infected hosts, such as febrile humans, cats, and dogs, where the normal body temperatures in the latter animal hosts can range from 38.3 to 39.2°C. This hypothesis aligns with the difficulty of isolating *B. henselae* from infected animals (Diniz et al., 2013; Sayed et al., 2022). Additionally, it suggests that fever in patients caused by *B. henselae* infection may also promote the transition to the VBNC state.

VBNC state has been proposed as part of bacterial persistence (Zhang, 2014; Ayrapetyan et al., 2018) for bacterial survival in adverse environments, exhibiting significant convergence in their characteristics and molecular mechanisms, particularly in antibiotic tolerance (Ayrapetyan et al., 2015b). Previous studies demonstrated that stationary-phase cultures contain a large portion of drug-tolerant persisters in various bacterial species, including *B. henselae* (Li et al., 2019). This study found that pure *B. henselae* VBNC cells exhibited enhanced drug tolerance relative to persisters (**Figure 2E**), especially against anti-persister drugs like daptomycin and methylene blue (Li et al., 2019). Currently, the VBNC state and bacterial persistence are regarded as closely related phenomena along a shared ‘dormancy continuum’, rather than entirely distinct states (Ayrapetyan et al., 2015b). However, the mechanisms underlying drug tolerance in *B. henselae* during these two forms may be different and require further investigation. High-temperature exposure also significantly accelerated the formation of *B. henselae* persisters (**Figure 2F**), suggesting that *B. henselae* in infected individuals may be more drug-tolerant, posing substantial challenges for clinical treatment.

Furthermore, clinically used antibiotics were shown to induce the VBNC state in *B. henselae*. Bacterial antibiotics like gentamicin and streptomycin eliminated culturable cells within 4 days (**Figure 3A**), which is consistent with our previous findings (Li et al., 2019). However, viable cell counts remained high. This, combined with successful resuscitation, unearthed a novel insight: these clinically employed antibiotics can induce *B. henselae* into VBNC state, at least under *in vitro* conditions. Even bacteriostatic antibiotics like doxycycline and erythromycin, also converted a portion of *B. henselae* cells to VBNC state (**Figure 3B**). Therefore, the current antibiotic treatment for *B. henselae*-infected individuals may have paradoxical effect of killing some growing bacteria while promoting VBNC cell formation, which can contribute to persistent and/or chronic infection. These findings advance our understanding of the impact of antibiotic treatment for *Bartonella* infections, urging the need to develop more effective treatments to combat the persister forms of this emerging human pathogen, especially the VBNC form.

The molecular mechanisms underlying VBNC formation in bacteria remain incompletely understood (Liu et al., 2023), though key genes and pathways, including those related to stress response, membrane proteins, toxin-antitoxin systems, and cell division, have been identified (Dong et al., 2020). Proteomic analysis in this study revealed that 17.6% of identified proteins were significantly altered in *B. henselae* VBNC cells compared to culturable cells. Notably, up-regulated proteins involved in hemin and iron uptake, such as FhaB1 and the hemin-binding proteins (Sander et al., 2000), suggest an iron deficiency in VBNC cells. This iron deficiency is supported by the resuscitation process, where sheep blood, rich in heme iron, promoted the recovery of VBNC cells to a culturable state. However, the specific contribution of sheep blood in the resuscitation process is a complex issue. Future studies should focus on identifying the key factors involved in the resuscitation of *B. henselae* VBNC cells, including the evaluation of various iron components.

Beyond their role in facilitating hemin and/or iron uptake, the HecB/FhaC proteins and hemin-binding proteins are also responsible for mediating *B. henselae* adhesion to host cells (Franz and Kempf, 2011). More proteins involved in host cell adhesion and invasion were up-regulated in VBNC cells (**Figure 4**; **Supplementary Table S2**; **Supplementary Figure S2**), including TrwL/J, VirB, and IalB. The Trw and VirB systems, which are Type 4 secretion system (T4SS), play critical roles in the adhesion of *Bartonella* to host cells (Jin et al., 2023), whereas IalB protein is implicated in erythrocyte invasion. The increased expression of these proteins in VBNC cells are likely responsible for facilitating the entry and invasion of *B. henselae* into host cells. FISH analysis in this study indeed confirmed that *B. henselae* in the VBNC state could invade HUVECs within 12-18 hours, whereas no invasion was observed for logarithmic-phase *B. henselae* during this same period (**Figure 4C**). Typically, actively growing bacteria require at least 24-30 hours to invade HUVECs (Dehio et al., 1997; Schmid et al., 2004). This suggests that VBNC *B. henselae* may have a faster invasion capability compared to its logarithmic-phase counterpart. This rapid invasion provides an effective strategy for countering environmental stress and evading the host immune clearance.

Another up-regulated protein in VBNC cells was BafA, a *Bartonella*-produced mitogen that exhibits pro-angiogenic activity through vascular endothelial growth factor receptor-2 (VEGFR-2) on the cell surface, resulting in the proliferation of *Bartonella*-infected vascular endothelial cells through activating the mitogen-activated protein kinase (MAPK) pathway (Tsukamoto et al., 2020). Other up-regulated proteins in VBNC cells included molecular chaperones and heat shock proteins (**Figure 4A**; **Supplementary Figure S2**), which are likely critical for maintaining the stability and folding of proteins in the VBNC state, thereby ensuring bacterial survival under unfavorable conditions. The down-regulation of cold shock proteins (**Figure 4A**) aligns with VBNC induction under high temperatures. SOD, an enzyme that eliminates reactive oxygen species, was also up-regulated, possibly aiding *B. henselae* VBNC cells survival at elevated temperatures.

Pathways involved in energy metabolism, lipid metabolism, and protein synthesis were up-regulated, while those related to the metabolism of nucleotides, amino acids, glycans, cofactors, and vitamins were down-regulated (**Figure 4B**). This metabolic shift suggests that *B. henselae* VBNC cells maintain essential functions for survival while down-regulating less critical pathways, characteristics of VBNC states (Liu et al., 2023) that share similarities with bacterial persister pathways (Zhang, 2014; Niu et al., 2024).

In conclusion, this study investigated *B. henselae*’s ability to enter the VBNC state and invade host cells efficiently. *B. henselae* can fully enter the VBNC state under fever-mimicking temperature (38.8°C) and antibiotic treatment, exhibiting enhanced drug tolerance and specific morphological and proteomic adjustments. The evidence suggests that bacteria in the VBNC state are not just a theoretical risk but may actively contribute to host pathogenesis, relapsing infections, and antibiotic failure. These findings underscore the diagnostic challenges posed by *B. henselae* in the VBNC state and emphasize the need for improving treatment strategies and developing effective culture methods to recover *B. henselae* isolates from patients.

## Data Availability

The original contributions presented in the study are publicly available. This data can be found here: ProteomeXchange website: http://proteomecentral.proteomexchange.org/cgi/GetDataset?ID=PXD055316 and iProX: https://www.iprox.cn//page/project.html?id=IPX0009557000.
