# Corrigendum: Viable but nonculturable state in the zoonotic pathogen *Bartonella henselae* induced by low-grade fever temperature and antibiotic treatment

**DOI:** 10.3389/fcimb.2024.1544596

**Published:** 2025-01-07

**Authors:** Yuze Gou, Dongxia Liu, Yuxian Xin, Ting Wang, Jiaxin Li, Yiwen Xi, Xiaoling Zheng, Tuanjie Che, Ying Zhang, Tingting Li, Jie Feng

**Affiliations:** ^1^ Key Laboratory of Preclinical Study for New Drugs of Gansu Province, School of Basic Medical Sciences, Lanzhou University, Lanzhou, China; ^2^ State Key Laboratory of Veterinary Etiological Biology, College of Veterinary Medicine, Lanzhou University, Lanzhou, China; ^3^ Department of Scientific Experimental Research, Innovation Center of Functional Genomics and Molecular Diagnostics Technology of Gansu Province, Lanzhou, China; ^4^ State Key Laboratory for the Diagnosis and Treatment of Infectious Diseases, The First Affiliated Hospital, Zhejiang University School of Medicine, Hangzhou, China; ^5^ Center for Microbiome and Disease Research, Jinan Microecological Biomedicine Shandong Laboratory, Jinan, China

**Keywords:** *Bartonella*, *B. henselae*, VBNC, resuscitation, persister, blood-culture-negative endocarditis (BCNE), cat-scratch disease (CSD)

In the published article, there was an error in affiliation 1. Instead of “Key Laboratory of Preclinical Study for New Drugs of Gansu Province, School of Basic Medical Sciences, Lanzhou, China”, it should be “Key Laboratory of Preclinical Study for New Drugs of Gansu Province, School of Basic Medical Sciences, Lanzhou University, Lanzhou, China”.

The authors apologize for this error and state that this does not change the scientific conclusions of the article in any way. The original article has been updated.

