# Supplementary material for: Viable but nonculturable state in the zoonotic pathogen Bartonella henselae induced by low-grade fever temperature and antibiotic treatment
Source: Front Cell Infect Microbiol. 2024 Nov 21;14:1486426. doi: 10.3389/fcimb.2024.1486426 (PMC11619046; doi:10.3389/fcimb.2024.1486426)
Supplement: Supplementary file 1 [file DataSheet1.docx]

**Supplementary**

**Table S1. MICs of drugs on *B. henselae* strain used in this study.**

| Drugs | MIC (μg/mL) |
| --- | --- |
| Ampicillin | 0.125-0.25 |
| doxycycline | 0.04-0.08 |
| ofloxacin | 4-8 |
| daptomycin | 6.25-12.5 |
| gentamycin | 0.31-0.63 |
| Erythromycin | 0.04-0.08 |
| Methylene blue | 0.08-0.16 |

**Table S2. The proteins specifically upregulated for HUVECs invasion.**

| Proteins | Name | Log2FC |
| --- | --- | --- |
| BH07920 | ShlB/FhaC/HecB_family_hemolysin_secretion/activation_protein | 1.28 |
| BH06540 | ShlB/FhaC/HecB_family_hemolysin_secretion/activation_protein | 3.80 |
| BH06660 | ShlB/FhaC/HecB_family_hemolysin_secretion/activation_protein | 2.38 |
| BH06550 | FhaB1 | 1.97 |
| BH02570 | Porin family protein | 3.33 |
| BH15670 | TrwJ | 2.35 |
| BH15700 | TrwJ | 1.88 |
| BH15570 | TrwL | 2.64 |
| BH15580 | TrwL | 2.54 |
| BH15590 | TrwL | 1.51 |
| BH15600 | TrwL | 1.65 |
| BH04390 | IalB | 0.55 |


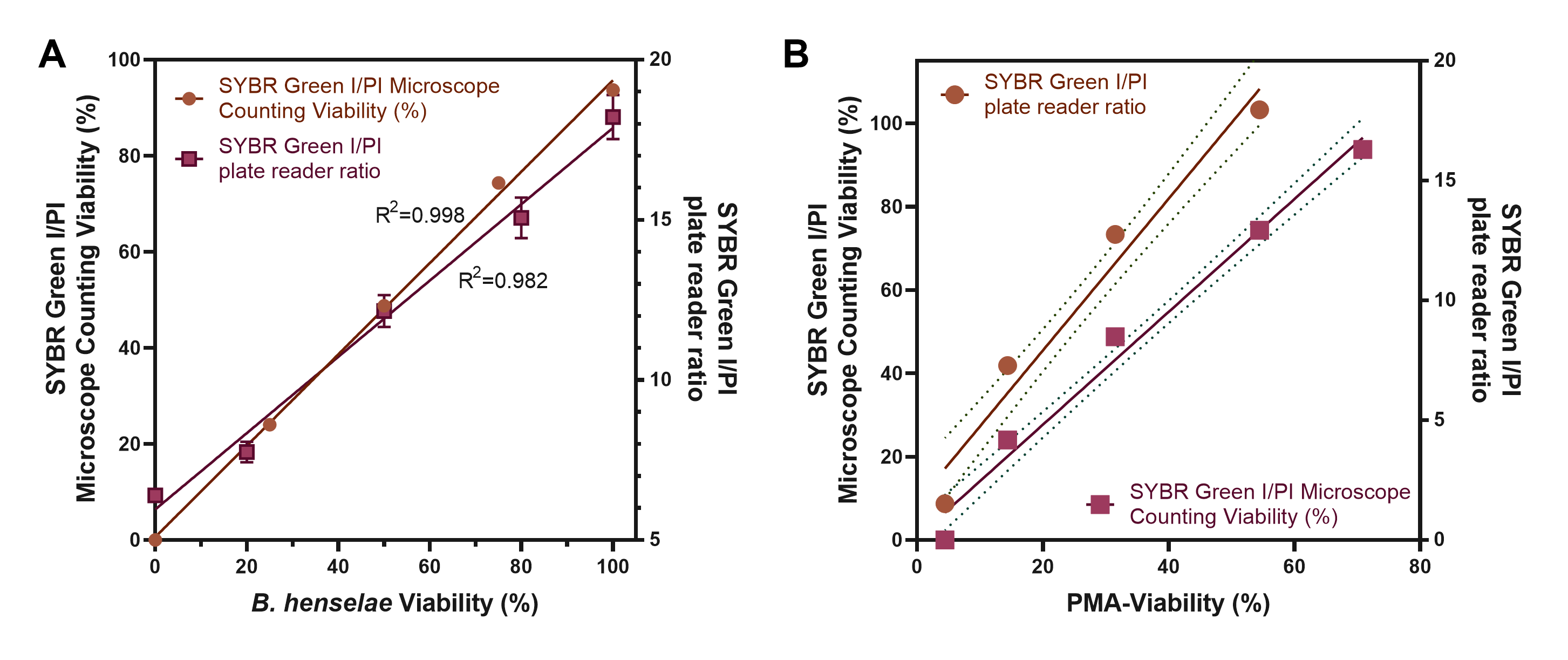


Figure S1. A. Liner regression analysis of the *B. henselae* viability and SYBR Green I/PI microscope counting or plate reader assay. B. Linear regression analysis of the PMA-viability assay and SYBR Green I/PI microscope counting or plate reader assay. All tests were run in triplicates, and mean values are shown.


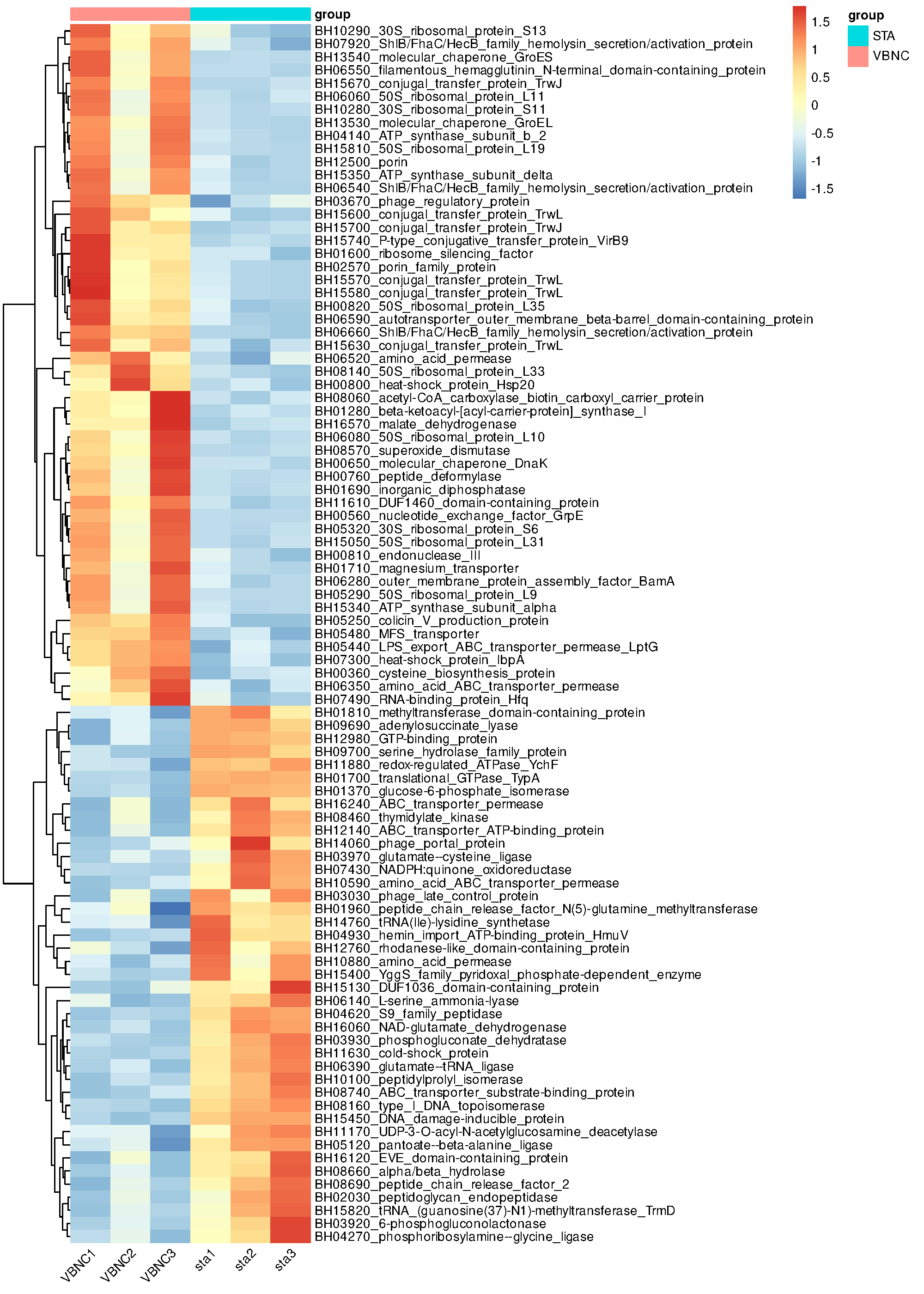


Figure S2. The heatmap shown the differentially expressed genes (*p*-value < 0.05 and Fold change > 2) of DIA proteomics analysis.
